# Dispersion and Preparation of Nano-AlN/AA6061 Composites by Pressure Infiltration Method

**DOI:** 10.3390/nano12132258

**Published:** 2022-06-30

**Authors:** Kai Sun, Ping Zhu, Pinliang Zhang, Qiang Zhang, Puzhen Shao, Zhijun Wang, Wenshu Yang, Dashuai Zhao, Martin Balog, Peter Krizik, Gaohui Wu

**Affiliations:** 1 Departmentof Materials Science and Engineering, Harbin Institute of Technology, Harbin 150001, China; 15776720450@163.com (K.S.); 18846450834@163.com (P.Z.); shaopuzhen@163.com (P.S.); hitzhijun@gmail.com (Z.W.); sunkingod@gmail.com (D.Z.); wugh@hit.edu.cn (G.W.); 2 Key Laboratory of Advanced Structure-Function Integrated Materials and Green Manufacturing Technology, Harbin Institute of Technology, Harbin 150001, China; 3 Beijing Institute of Spacecraft Environment Engineering, Beijing 100094, China; zhangpinliang620@126.com; 4 Institute of Materials and Machine Mechanics, Slovak Academy of Sciences, Dubravska Cesta 9, 84513 Bratislava, Slovakia; ummsbama@savba.sk (M.B.); peter.krizik@savba.sk (P.K.)

**Keywords:** nano-AlN, MMC, pressure infiltration, dispersion, hot extrusion, mechanical properties

## Abstract

Nanomaterials play an important role in metal matrix composites (MMC). In this study, 3.0 wt.%, 6.0 wt.%, and 9.0 wt.% nano-AlN-particles-reinforced AA6061 (nano-AlN/AA6061) composites were successfully prepared by pressure infiltration technique and then hot extruded (HE) at 500 °C. The microstructural characterization of the composites after HE show that the grain structure of the Al matrix is significantly refined, varying from 2 to 20 μm down to 1 to 3 μm. Nano-AlN particles in the composites are agglomerated around the matrix, and the distribution of nano-AlN is improved after HE. The interface between AA6061 and nano-AlN is clean and smooth, without interface reaction products. The 3.0 wt.% nano-AlN/AA6061 composite shows an uppermost yield and supreme tensile strength of 333 MPa and 445 MPa, respectively. The results show that the deformation procedure of the composite is beneficial to the further dispersion of nano-AlN particles and improves the strength of nano-AlN/AA6061 composite. At the same time, the strengthening mechanism active in the composites was discussed.

## 1. Introduction

With its light weight, high distinct strength and stiffness, excellent abrasion resistance, and tailorable expansion coefficient, Al-based composite materials (Al-MMC) have the potential to be applied in the aerospace, automotive, and military industries [1,2,3]. For the past few years, with the rapid development of nanomaterials such as graphene and carbon nanotubes (CNTs) [4], nano-Al-based composite materials [5,6,7] have received widespread attention from materials scientists. In addition, some nanomaterials such as AlN are also very important for nuclear fusion technology [8]. Some researchers have shown that the size of the reinforcing phase is one of the major aspects affecting mechanical properties of composites [9]. According to the current research, the addition of a small amount of nano-reinforcing phase can control the structure and further improve the performance of MMC [10,11,12]. Zhang et al. [6] synthesized CNTs/Al composites by flake powder metallurgy (PM) and nanometer Al_2_O_3_ formed in situ, the tensile strength of which is 79.5% higher than that of the Al matrix. However, because of the difference in physical properties between the nano-reinforcement and the matrix, the agglomeration of the nano-reinforcement has been always an essential problem for nano-reinforced MMCs [13,14,15]. To settle the issue, a series of dispersion methods of nano-reinforcements has been introduced, such as molecular-level mixing [16], flake powder metallurgy [17], etc. Hwang et al. [18] used molecule-level mixing to attach functional groups to graphene flakes and chemically bind graphene to a composite matrix, engendering a uniform dispersion of graphene within the matrix. Lin et al. [19] utilized the flake powder metallurgy method of slurry blending to adsorb CNTs in Al nanosheets, which solves the problem of incompatibility between Al powder and CNTs and made the uniform distribution of CNTs in Al powder. At the same time, subsequent deformation treatments such as extrusion, rolling, etc., to prepare the composite material contribute to the nano-reinforcement dispersed in the matrix. Chen et al. [20] used hot press sintering and HE to make 5 wt.% AlN/Mg composites. These dispersion methods of nano-reinforcement can also be used as a reference for the dispersion of other nano-reinforcements.

Aluminum nitride (AlN) with its outstanding mechanical properties, low thermal expansion coefficient, and thermal stability has attracted the attention of many researchers [21,22]. At the same time, some characterization methods of aluminum nitride came into being, such as cathodoluminescence measurements (CL), X-ray absorption near edge spectroscopy (XANES), and Fourier-transform infrared spectroscopy (FTIR), which promoted the further development of AlN [23]. However, pure AlN materials are inherently brittle and are difficult to produce on a large scale and at low cost, so the field of application and commercialization are severely limited [24,25,26]. So, if AlN is employed as a reinforcement of AMC, the advantages of it can be supplemented to prepare low density and high-strength composites. AlN has stable chemical properties and does not react with the Al matrix. In this way, a degradation in the composite’s properties caused by poor interface reaction can be avoided. Therefore, using AlN as a reinforcement is expected to produce lightweight, high-strength, and low-thermal-expansion AMC. Wang et al. [27] produced the AlN/Al composites by cold isostatic pressing and then HE. The tensile strength reached 310 MPa, which was four times higher than that of pure Al. At the same time, the thermal expansion coefficient was reduced to 14.3 × 10^−6^. Tang et al. [28] prepared AlN nanowire/Al composites with uniform distribution by vacuum hot pressing, and their maximum tensile strength was about five times that of the Al matrix. Xu et al. [29] prepared AlN and Si_3_N_4_ particles to reinforce Al matrix composites through a liquid–solid reaction process, and the tensile strength of the composites prepared by adding 12.7% of the reinforcement was four times that of pure Al. However, due to the low wettability between AlN and the Al matrix, the interface bonding of the composite material is poor, which affects the further improvement in the composite’s material properties. Compared with the traditional powder metallurgy method, the pressure infiltration method can infiltrate the molten Al into the preform to avoid the problem of poor wettability between the matrix and the reinforcement. At present, composite materials with AlN as their reinforcement mainly use pure Al as the matrix, and there are few studies on the use of an Al alloy as the matrix. AA6061 has high strength and plasticity and good processing performance. Therefore, replacing pure Al with AA6061 as the matrix is better to improve the strength of materials.

In this research, flake AA6061 powder was produced through a ball milling step in two steps, and nano-AlN was uniformly dispersed on the surface of the AA6061 powder. The pressure infiltration process was adopted to solve the problem of the poor wettability between Al and nano-AlN, and nano-AlN/AA6061 composites with an appealing performance were prepared. After that, the composite was deformed by HE to further improve the distribution of nano-AlN particles in the matrix in order to further increase the mechanical properties of composites. The microstructure and mechanical properties of the produced Al-MMC, as well the calculation of active strengthening, mechanisms are pursued.

## 2. Materials and Methods

### 2.1. Materials and Praperation

**Figure 1:**
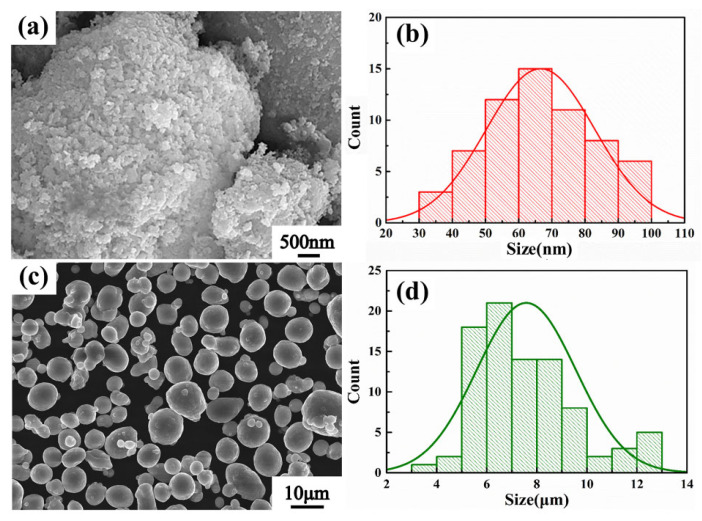
SEM images of (**a**) nano-AlN and (**c**) AA6061 powders. (**b**,**d**) The particle size histograms of nano-AlN and AA6061 powders, respectively.

The nano-AlN particles (Figure 1a) were supplied by the Shanghai Yao Tian Nanomaterials Technology Co. Ltd., Shanghai, China, and has a mean particle size (d50) of 70 nm. The gas-atomized AA6061 powder (Figure 1c) with a mean particle size (d50) of 7.5 μm was supplied by Northeast Light Alloy Corp. Ltd., Harbin, China. The chemical composition of AA6061 is displayed in Table 1.

**Figure 2:**
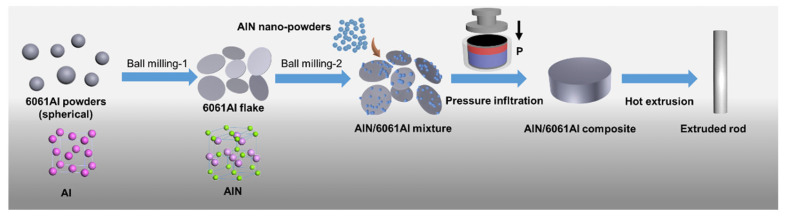
The schematic preparation process of nano-AlN/AA6061 composite.

The schematic preparation process is shown in Figure 2. Both ball milling-1 and ball milling-2 steps used the same process conditions. Both were conducted using a planetary mill in zirconia ball milling tanks with a rotation speed of 200 rpm for 6 h. We used zirconium oxide balls with a diameter of 6 mm as the milling medium (ball to powder weight ratio of 10:1). In the step of ball milling-1, the shape of the AA6061 changed from spherical to flake-like morphology. Afterwards, we added nano-AlN particles during the ball-milling-2 step (the weight ratio of the nano-AlN in the Al powder was 3 wt.%, 6 wt.% and 9 wt.%). Then, we put the mixture into the steel mold and pressed it to the predefined height in order to obtain the preforms. The volume content of milled particles in the mold was about 60 vol.% and a final residual porosity in the preform (40%). After that, the perform and pressure infiltration dye was put into the stove at 550 °C and 760 °C for preheating. In the infiltration process, the pressure of 10 MPa was used and kept for 7 min, followed by the solidification process in the air, and then the nano-AlN/AA6061 composites were obtained. Afterward, the nano-AlN/AA6061 composites were HE at 500 °C with an extrusion ratio of 13:1. All samples were solid solution treated in a salt bath furnace at 530 °C for 1 h and the aged at 175 °C for 6 h before the microstructure investigation and mechanical properties test.

### 2.2. Characterization Methods

Morphologies of the nano-AlN/AA6061 composites were investigated through the Olympus PMG3 light microscop (Olympus Corporation, Tokyo, Japan). Morphologies of the nano-AlN, AA6061 powders, and the nano-AlN/AA6061 composites were characterized by FEI Quanta 200FEG scanning electron microscope (SEM) (Waltham, MA, USA). X-ray diffraction (XRD) analysis was performed by using Rigaku D/max-RB diffractometer (Rigaku Corporation, Tokyo, Japan). The specimens were subjected to Cu-Kα radiation (0.15418 nm) with a scanning speed set at 2°/min while the 2θ scans were tested from 20° to 90°. TEM investigation was tested on Talos f200x (FEI, Hillsboro, OR, USA). Tensile tests were conducted using an Instron 5569 universal tensile testing machine (Instron, Boston, MA, USA) with a cross-head speed of 0.1 mm/min. For the accuracy of the test, every test was performed at least four samples. The fracture surface of the nano-AlN/AA6061 composites after tensile test was also observed by FEI Sirion Quanta 200 SEM.

## 3. Results and Discussion

### 3.1. Microstructure of Nano-AlN/AA6061 Composites

**Figure 3:**
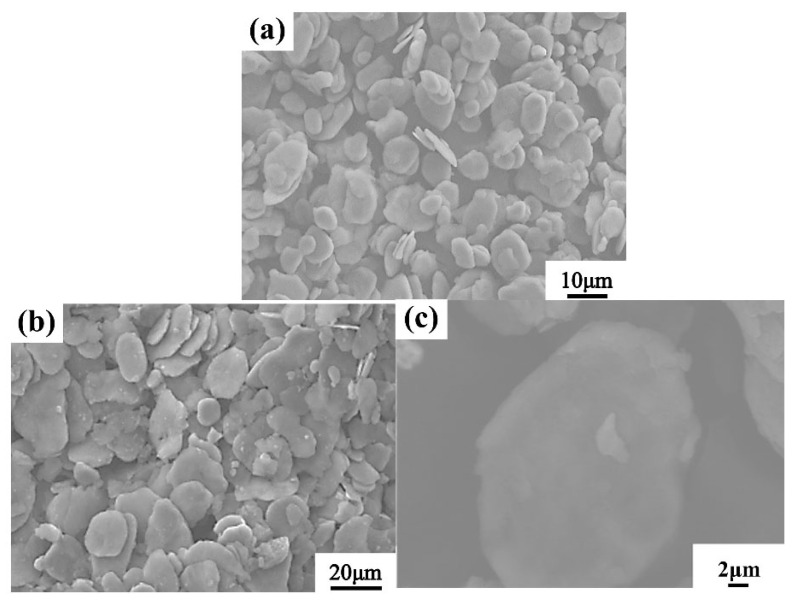
SEM images of (**a**) AA6061 flakes prepared after ball milling-1; (**b**,**c**) Nano-AlN/AA6061 flakes prepared after ball milling-2.

Two ball milling steps are widely used to change the morphology of Al powders. It can be clearly seen in Figure 3a that the spherical AA6061 powder is transformed into flakes after ball milling-1, and the surface area of the Al powder increased in order to facilitate the dispersion of nano-AlN particles. Figure 3b,c show the morphologies of the mixed powder after ball milling-2. During this step, the Al still remains in the form of flakes, but the flake size increases. This process consists of two effects. The first effect is to undergo a flake formation of the AA6061 matrix, and the second one is to disperse the nano-AlN particles under the behavior of high-energy ball milling on the surface of the substrate to make a powder mixture.

In the process of creating high-energy ball milling powder, we control the cold-welding phenomenon of the powder by adding alcohol as a process control agent, which can improve the ball milling efficiency and avoid the problem of nano-AlN agglomerating during ball milling. At the same time, by adding the process control agent, the surface of the powder tends to be smoother.

**Figure 4:**
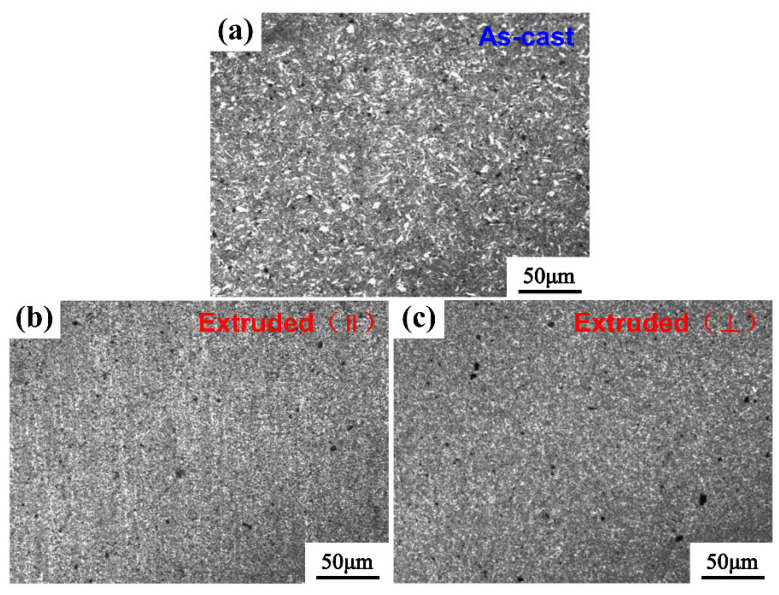
SEM images of nano-AlN/AA6061composite: (**a**) as-cast; (**b,c**) after HE(the extrusion direction is marked in the figure).

Table 2 displays the density and relative density of nano-AlN/AA6061 composites in different states. We can see that the relative densities with different mass fractions are all increased after HE. SEM images of as-cast and hot-extruded 3 wt.% nano-AlN/AA6061 composites with the extrusion direction marked are shown in Figure 4. It can be seen from Figure 4a that the structure of the as-cast composite is uniform. Some pores can be still seen in the picture, which causes the density of the material to decrease. Figure 4b demonstrates that the morphologies of the nano-AlN/AA6061 composites are more uniform, and the number and size of the holes have decreased significantly after HE. The impregnated Al liquid is completely refined, but a few pore defects can still be observed, indicating that HE does not eliminate enough of the residual porosity in as-cast materials. Meanwhile, it can be seen that the arrangement of the reinforcement is parallel to the extrusion direction Figure 4c, which can clearly distinguish the arrangement between the ball-milled AA6061 powder and the impregnated Al liquid. In the as-cast material, a relatively coarse structure was observed in the composite material, and the composite material after HE was considerably refined to form a dense structure with a regular arrangement. The nano-AlN benefits from the arranged uniform and refined microstructures in terms of improved strength [30,31].

**Figure 5:**
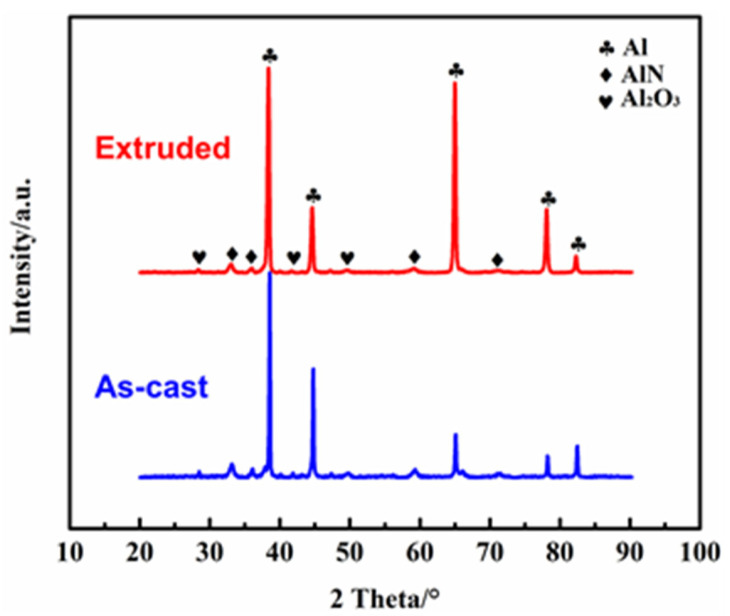
XRD patterns of nano-AlN/AA6061 composite.

Figure 5 displays the XRD pattern of the 3 wt.% nano-AlN/AA6061 composite material in as-cast and as-extruded conditions. The XRD patterns of the composites with 6.0 wt.% and 9.0 wt.% contents show similar XRD lines. It can be seen that there are diffraction peaks in the AA6061 matrix and in the reinforcement of AlN in the composite materials. We can also see that the intensity of the diffraction peaks of AlN increases with the increase in the content of nano-AlN particles. At the same time, the peak in Al_2_O_3_ is found in the figure, which indicates that the AA6061 matrix in the composite material reacts with oxygen in the air under the atmospheric environment to form an oxide layer on the surface [32].

### 3.2. Distribution of Nano-AlN Particles

**Figure 6:**
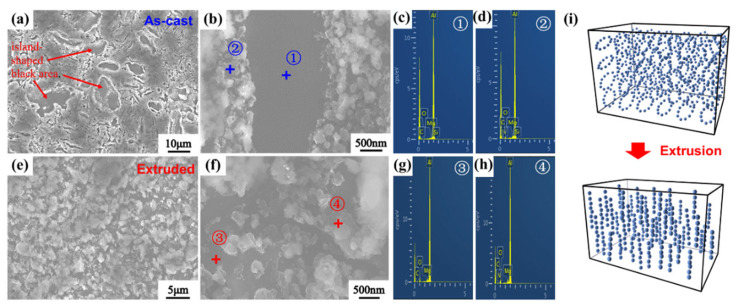
SEM images of nano-AlN/Al composites after electrolytic etching: (**a**,**b**) As-cast; (**e**,**f**) After HE. (**c**,**d**,**g**,**h**) are the energy spectrum of points 1, 2, 3, and 4. (**i**) is the schematic diagram of distribution of AlN particles before and after HE.

The images of the as-cast and extruded composite materials after electrolytic etching experiments are shown in Figure 6. From Figure 6a, it can be seen that the as-cast nano-AlN/Al composites after etching show two distinct areas: a gray-white continuous area and an island-shaped black area. The infiltrated liquid Al presents a gray-white continuous area after etching, and the morphology and size of the island-like black area are similar to the flaky shape of the powder after two steps ball milling (Figure 3b), with a grain size distribution between 2 and 10 μm. In addition, there is a distinct granular substance at the junction of the two regions. To further determine the composition of the island-like black region and the granular material, we performed an elemental analysis of 1 and 2 points in Figure 6a using the energy spectrum. It can be obtained from Figure 6c that the main element distribution of the island-shaped black region comprises Al, Mg, and Si, which are the main alloying elements in AA6061 alloy. Therefore, it is assumed that the composition of the island-shaped region is mainly AA6061, which is a morphology exhibited by the flake-shaped AA6061 [33]. Figure 6d displays the energy spectrum elements on point 2; it shows a more obvious N element distribution. Therefore, it can be concluded that the nano-AlN particles surround the discrete AA6061 powder flakes after ball milling. From Figure 6e,f of the vertical extrusion direction of the composite material after electrolytic etching, the area of the flaky AA6061 substrate and the area of the uncorroded liquid AA6061 can be seen. Unlike the black flaky areas found in the as-cast material after etching, the black areas in the material after HE are more detailed, with a large number of small areas, showing a semi-continuous state. According to the spectroscopic point analysis of Figure 6g,h, no N element was found in the lamellar AA6061 matrix region, and the presence of N element was obtained in the liquid AA6061 region, indicating that the nano-AlN particles are arrayed. After the pressing treatment, nano-AlN particles are still distributed on the surface of the flaky AA6061 substrate. With the refinement of the flaky AA6061 substrate, the dispersibility of the nano-AlN particles improves further. The AlN changed from being uniformly arranged, as shown in Figure 6i, to being oriented along the extrusion direction.

**Figure 7:**
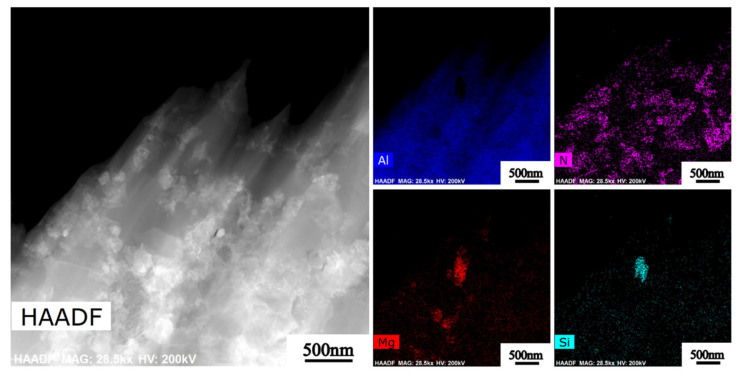
HAADF images of nano-AlN/Al composite after HE.

**Figure 8:**
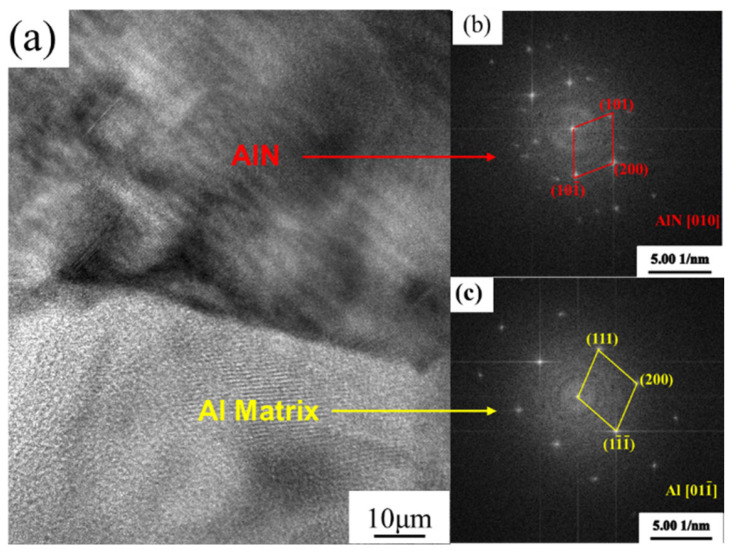
HRTEM of nano-AlN/AA6061 after HE: (**a**) Interface of nano-AlN and AA6061; (**b**,**c**) the FFT result of the selected region.

Figure 7 and Figure 8 show a high-resolution image of the matrix, precipitated phase, and nano-AlN interface area of the composite material and the calibration of the diffraction spots. From Figure 7, it can be seen that there is an obvious precipitated phase of Mg_2_Si in the Al matrix. However, the speckle calibration found only the spots of Al, and no obvious precipitated phase spots were found. This may be explained by the size of the precipitated phase being too small. Moreover, another reason may be that Mg is consumed when it binds with O, resulting in insufficient Mg binding with Si. Therefore, the precipitation phase with an Mg/Si ratio less than 2 is incorporated in the material. According to Figure 8 it can be seen that the interface between the AA6061 and the nano-AlN is smooth, flat, and without obvious holes, which indicates that a good interface bonding has been obtained between the nano-AlN particles and the AA6061. In addition, there are no interface reaction products. This compact interface helps to increase the mechanical properties of the nano-AlN/AA6061 composites.

### 3.3. Machinal Properties of Nano-AlN/AA6061

**Figure 9:**
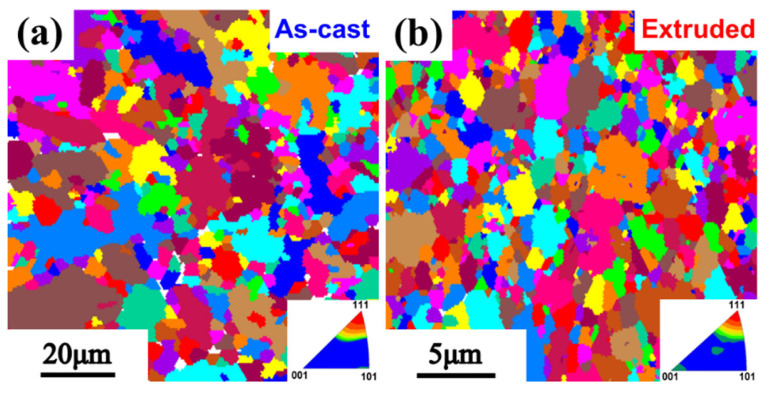
EBSD images of nano-AlN/AA6061 composite: (**a**) As-cast; (**b**) After HE.

The grain sizes of the as-cast composites and the composites after HE are shown in Figure 9. After HE treatment, the average grain size of the AA6061 in the composites has been extremely refined. The grain size of the as-cast material is mainly distributed between 2 and 20 μm, and the grain sizes of the composites after HE are mainly distributed between 1 and 3 μm. It has been widely noted in the Al matrix composites that the hot-extruded treatment has an obvious effect of grain refinement, developing the interfacial bonding and directional arrangement of the reinforcements which are valuable for the improvement in the mechanical properties of the composites.

**Figure 10:**
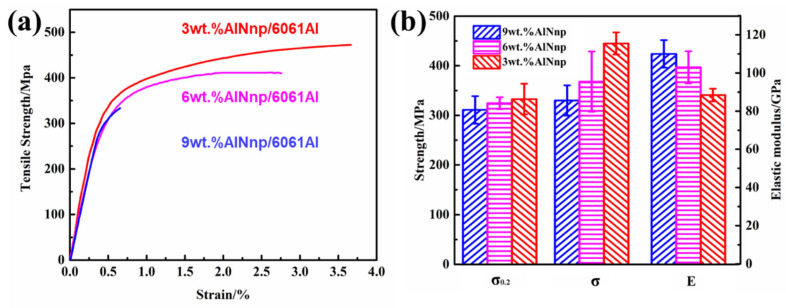
Stress–strain curves of nano-AlN/AA6061 composites: (**a**) Tensile curve; (**b**) Comparison of yield strength, tensile strength, and elastic modules.

Figure 10 presents the tensile curves of nano-AlN/AA6061 composites after HE with 3 wt.%, 6 wt.%, and 9 wt.% nano-AlN, respectively. It is observed from the curve that, with the content of the nano-reinforcement increases, the yield strength, tensile strength, and elongation of the corresponding composite material tensile curve shows a downward trend. The elastic module of the 3 wt.%, 6 wt.%, and 9 wt.% nano-AlN/AA6061 composites were 88 GPa, 103 Gpa, and 110 Gpa, respectively. Moreover, they showed an upward trend with the increase in the nano-reinforcement content. The specific performance parameter comparison chart is presented in Figure 10b. It illustrated that the average yield strength of the 3 wt.%, 6 wt.%, and 9 wt.% nano-AlN/AA6061 composite materials are 333 Mpa, 324 Mpa, and 311 Mpa, respectively. Compared to the 3 wt.% AlNnp/AA6061 materials, 6 wt.% and 9 wt.% nano-AlN/AA6061 composites have yield strengths that decrease by 2.7% and 6.6%, respectively. The difference in yield strength between the three composites is not particularly significant. The tensile strength of the three composite materials was 445 Mpa, 368 Mpa, and 330 Mpa, respectively. The tensile strength of the 6 wt.% and 9 wt.% nano-AlN/AA6061 composite materials were reduced by 17% and 36%, respectively. The elongations of the three composites were 2.8%, 1.7%, and 0.52%, respectively. The elongation of the 6 wt.% and 9 wt.% nano-AlN/AA6061 composites decreased by 39% and 81%, respectively. The biggest influencing factor is that during the two-step ball milling dispersion process, as the content of the nanoparticles rises, the difficulty in achieving uniform dispersion also increases [34]. AlN agglomerates in the matrix and is not dispersed during composite preparation. Therefore, the yield strength of the three composites is equivalent, and there is no significant difference, but plastic deformation occurs at the beginning of yielding. In the process of deformation, because of the uncoordinated deformation between the agglomerated nano-AlN particls and the AA6061 matrix, as well as defects such as holes, the composite materials form crack sources and expand rapidly under tensile action, resulting in fracture, lower tensile strength, and lower plasticity. Meanwhile, the average yield strength and tensile strength of the AA6061 alloy prepared using the same process are 321 Mpa and 362 Mpa, respectively.

**Figure 11:**
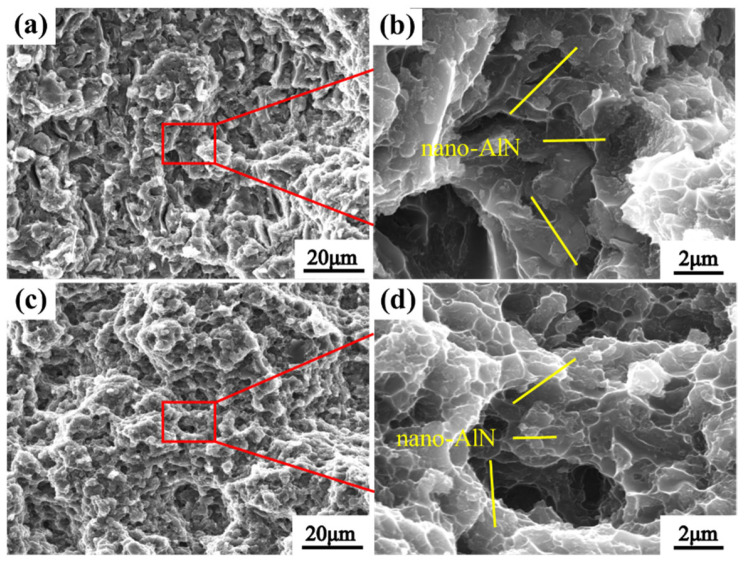
Fracture surface of nano-AlN/Al composites: (**a**,**b**) As-cast; (**c**,**d**) After HE.

Figure 11 shows the fracture surface of the of-cast and HE nano-AlN/AA6061 composites. We can see from the low-magnification image in Figure 11a that there are some hole defects in the as-cast composites. After HE treatment, the number of voids decreased obviously, the fracture surface was uneven, indicating that the fracture process must take a certain path, and the matrix was observed to be torn, which is beneficial to the exertion of plasticity. The presence of particles and precipitates in the dimples can be seen in the enlarged image. It is generally believed that the formation of dimples is the result of micropore growth. [35,36,37]. From the illustration, the micropores are mostly concentrated in the second-phase particles and the precipitates. Comparing the number of dimples of the fracture before and after HE, there is an increased number of dimples in the material after HE. The sizes of the AlN particles are mainly between 1 and 3 nm, and the AlN nanoparticles are found in the fracture surface and dimple sidewall. Because the elastic–plastic deformation of the nano-AlN particles is different from that of matrix AA6061, micropores are formed in the interface cracks between them. Therefore, it is obvious that nanoparticles can induce micropores to form dimples. [38]. Poles tend to expand towards them, accelerating the propagation of cracks and causing fractures [39].

### 3.4. Strengthening Mechanism of Nano-AlN/AA6061

In this part, we take the composites with 3% nano-AlN as an example to discuss the strengthening mechanism. The possible strengthening mechanisms of the particle-reinforced composites have been argued in earlier studies: the load transfer from the Al matrix to reinforcements ($\Delta \sigma_{L}$) in [40]; grain refinement results from the pinning effect of AlN particles ($\Delta \sigma_{G}(R)$) and hot extrusion in [10]; the thermal mismatch mechanism is caused by the generation of dislocations due to the different coefficients of the thermal expansion (CTE) between the Al matrix and the reinforcements ($\Delta \sigma_{T}$) in [41]; and the Orowan strengthening mechanism is attributed to the Orowan looping system ($\Delta \sigma_{oro}$). Thus, the multiple strengthening mechanisms operating in the composite can be expressed as in Equation (1):

(1) $$\sigma_{c}=\sigma_{m}+\Delta \sigma_{G}+\Delta \sigma_{T}+\Delta \sigma_{oro}+\Delta \sigma_{L}$$

The strengthening influences by grain refinement can be calculated through the Hall–Petch formula using Equation (2):

(2) $$\Delta \sigma_{G}(R)=K(d_{1}{}^{-1/2}-d_{0}{}^{-1/2})$$

where *K* is a constant for Al, which is 0.065 Mpa·m^1/2^ [42,43,44]. *d* is the average grain size of the composite after hot extrusion, as shown in Figure 9, and it can be measured as 2.6 μm. So, the results show that the contribution of the grain refinement effect is about 7.5 Mpa. This effect is mainly related to the inhibition of the reinforcement particles on grain grown during the preparation process and the hot extrusion. Meanwhile, for the CTE differences between the reinforcement and matrix, a number of dislocations can be generated from the interface during the sodification process at the end of the hot extrusion. The new dislocations make a significant contribution to the composites [45]. The CTE of AlN is 4.5 × 10^−6^ and 23.6 × 10^−6^ for the matrix Al. The contribution of thermal mismatch mechanism to tensile strength can be assessed by the following formulas [46]:

(3) $$\Delta \sigma_{T}=USS/0.65-\sigma_{m}$$

(4) $$USS=\tau_{m}+kG_{m}b\sqrt{\rho }$$

where *USS* is the ultimate shear strength of the composites. k is a constant (0.5), and b is the Burgers vector of matrix Al (0.286 nm). $G_{m}$ is the shear modulus of matrix and can be calculated using the basic parameters of Al. According to the literature, $G_{m}$ is about 27.5 Gpa [6,47]. It is worth noting that the enhanced dislocation density (ρ) in the composite may be calculated by Equation (5):

(5) $$\rho =12\frac{\Delta \alpha \Delta TV_{R}}{d_{R}b(1-V_{R})}$$

where Δα is the difference in CTE between the two parts, the matrix and the reinforcement. ΔT is the gradient in the temperature from hot extrusion (500 °C) to the ambient temperature (25 °C). $V_{R}$ and $d_{R}$ are the volume fraction and approximate diameter of the reinforcements, respectively. $d_{R}$ can be obtained from Figure 1. According to the Equation (3), the contrition of the thermal mismatch mechanism is about 45.4 Mpa.

The particles in the composites can inhibit the movement of dislocation, and this may enhance the strength of the materials. This mechanism plays an important part in strengthening the material, which may be described by the Orowan formula (Orowan-Ashby equation [48]), as in Equation (6):

(6) $$\Delta_{Oro}=\frac{0.13G_{m}b}{\lambda_{R}}\ln\frac{r_{R}}{b}$$

(7) $$\lambda_{R}\approx \frac{d_{R}\left[{\left(\frac{1}{2V_{R}}\right)}^{1/3}-1\right]-d_{R}}{\cos\theta }$$

where $G_{m}$ and b are the shear modulus and the Burgers vector of matrix Al, as mentioned before, while $r_{R}$ is the radius of the nano-AlN, which is half the diameter ($d_{R}$). θ is the angle between the nano-AlN and the dislocation, simplifying that the minimum angle (0°) and the maximum angle (90°) correspond to the cases of dislocation travelling parallel and vertically, respectively, in relation to the reinforcement [49]. $\lambda_{R}$ is the effective inter-particle spacing caused by the different alignment between nano-AlN and AA6061 and can be calculated by Equation (7). $V_{R}$ is the volume fraction of reinforcements in composite. The result indicates that the contribution from Orowan looping system is about 22.6 Mpa.

**Figure 12:**
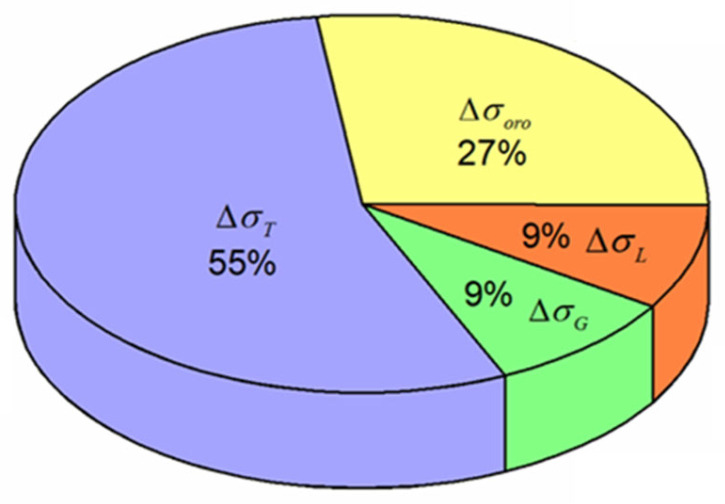
The proportion of each strengthening mechanism in nano-AlN/AA6061 nanocomposites.

Through calculation, it can be concluded that the strengthening effect of the load transfer is about 7.5 Mpa. Figure 12 shows the proportion of each strengthening mechanism. From the above results, it can be seen that thermal mismatch strengthening is the most important contribution.

**Figure 13:**
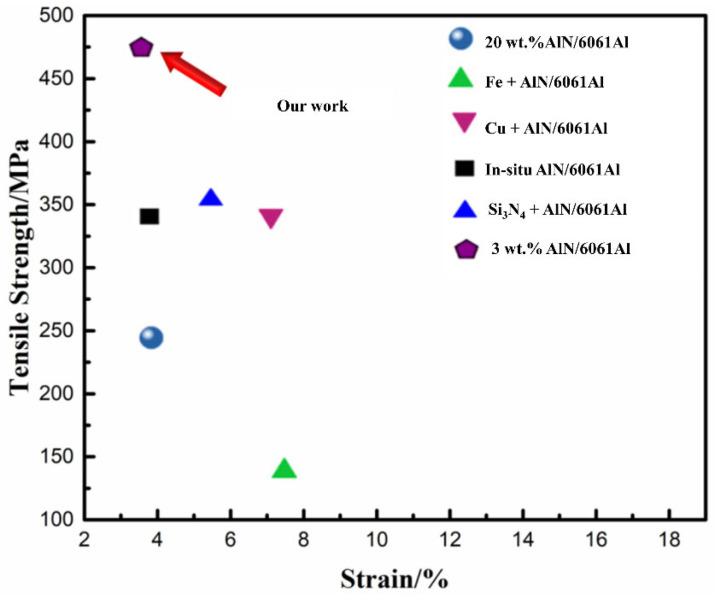
Comparison of the yield strength of nano-AlN/AA6061 composites in literature [49,50,51,52].

As shown in Figure 13, compared with the micro-AlN used in the literature, the composites prepared by sheet powder metallurgy, pressure infiltration and hot extrusion have higher mechanical properties. Selecting the appropriate ball milling process, the appropriate content of reinforcement and the hot extrusion deformation can improve the structure of the composite and eliminate the agglomeration of nanoparticles, which is beneficial to improve the reinforcement efficiency. In addition, compared with the traditional particle reinforcement, nano-reinforcement shows obvious advantages.

## 4. Conclusions

The Al composites with a uniform distribution of AlN reinforcement were successfully prepared by the pressure infiltration process. After HE, the AA6061 matrix grains were refined, and the reinforcement distribution was found arrayed along the extrusion direction. Meanwhile, the size of Al grains decreased from 2 to 20 μm to 1 to 3 μm and the preferred orientation was found in the direction <111>. The interface between Al and AlN was metallurgically clean with no interfacial reaction products formed. The tensile strength of the 3.0 wt.% nano-AlN/Al composite material reached up to 445 Mpa, and the elongation up to 3.6% after HE and T6 treatment. According to the calculations of the active strengthening mechanisms, the thermal mismatch, Orowan, and load transfer mechanism all play a major role.

## Figures and Tables

**Table 1:** Chemical compositions of AA6061 powder.

Element	Mg	Si	Cu	Fe	Zn	Al
Wt.%	1.12	0.8	0.3	0.7	0.25	Bal.

**Table 2:** Density and relative density of nano-AlN composites of as-cast and HE.

Materials	Status	Density (g cm^−3^)	Relative Density (%)
3 wt.%nano-AlN/AA6061	As cast	2.40	88.1
	Heat extruded	2.60	95.4
6 wt.%nano-AlN/AA6061	As cast	2.34	85.2
	Heat extruded	2.53	92.1
9 wt.%nano-AlN/AA6061	As cast	2.26	81.5
	Heat extruded	2.49	89.8

## Data Availability

Data presented in this article are available at request from the corresponding author.
